# The influence of spirituality on decision-making in palliative care outpatients: a cross-sectional study

**DOI:** 10.1186/s12904-020-0525-3

**Published:** 2020-02-21

**Authors:** Francisca Rego, Florbela Gonçalves, Susana Moutinho, Luísa Castro, Rui Nunes

**Affiliations:** 1 Faculty of Medicine, University of Porto, Alameda Prof. Hernâni Monteiro, 4200-319 Porto, Portugal; 2 Portuguese Institute of Oncology-Coimbra, Instituto Português de Oncologia de Coimbra Francisco Gentil, E.P.E., Av. Bissaya Barreto 98, 3000-075 Coimbra, Portugal; 3 Portuguese Institute of Oncology-Porto, Instituto Português de Oncologia do Porto Francisco Gill E.P.E., Rua Dr. António Bernardino de Almeida, 4200-072 Porto, Portugal; 4 Institute for Systems and Computer Engineering, Technology and Science, INESCTEC, Rua Dr. Roberto Frias, 4200-465 Porto, Portugal; 5 Center for Health Technology and Services Research – CINTESIS, University of Porto, Rua Dr. Plácido da Costa, 4200-450 Porto, Portugal

**Keywords:** Autonomy, Decision-making, Decisional conflict, Palliative care, Spirituality, Spiritual wellbeing

## Abstract

**Background:**

Decision-making in palliative care can be complex due to the uncertain prognosis and general fear surrounding decisions. Decision-making in palliative care may be influenced by spiritual and cultural beliefs or values. Determinants of the decision-making process are not completely understood, and spirituality is essential for coping with illness. Thus, this study aims to explore the influence of spirituality on the perception of healthcare decision-making in palliative care outpatients.

**Methods:**

A cross-sectional study was developed. A battery of tests was administered to 95 palliative outpatients, namely: sociodemographic questionnaire (SQ), Decisional Conflict Scale (DCS), Functional Assessment of Chronic Illness Therapy-Spiritual Well-Being scale (FACIT-Sp), and a semi-structured interview (SSI) to study one’s perception of spirituality and autonomy in decision-making. Statistical analyses involved descriptive statistics for SQ and SSI. The Mann-Whitney test was used to compare scale scores between groups and correlations were used for all scales and subscales. The analysis of patients’ definitions of spirituality was based on the interpretative phenomenological process.

**Results:**

Spiritual wellbeing significantly correlated with greater levels of physical, emotional and functional wellbeing and a better quality of life. Greater spiritual wellbeing was associated with less decisional conflict, decreased uncertainty, a feeling of being more informed and supported and greater satisfaction with one’s decision. Most patients successfully implemented their decision and identified themselves as capable of early decision-making. Patients who were able to implement their decision presented lower decisional conflict and higher levels of spiritual wellbeing and quality of life. Within the 16 themes identified, spirituality was mostly described through family. Patients who had received spiritual care displayed better scores of spiritual wellbeing, quality of life and exhibited less decisional conflict. Patients considered spirituality during illness important and believed that the need to receive spiritual support and specialised care could enable decision-making when taking into consideration ones’ values and beliefs.

**Conclusion:**

The impact of spiritual wellbeing on decision-making is evident. Spirituality is a key component of overall wellbeing and it assumes multidimensional and unique functions. Individualised care that promotes engagement in decision-making and considers patients’ spiritual needs is essential for promoting patient empowerment, autonomy and dignity.

## Background

Palliative care is a patient-centred, transcultural and holistic approach that is essential to address the patient as a whole. In order to understand the multifaceted experience of suffering, Cicely Saunders developed the concept of total pain, which encompasses the physical, social, psychological and spiritual dimensions of end of life patients [1]. The biopsychosocial-spiritual model was also developed as an expansion upon the biopsychosocial model, which considers the spiritual concerns of patients and makes healthcare professionals aware of the need to attend these issues [2, 3].

Spirituality is a significant dimension of quality of life along with physical and psychological wellbeing in palliative care patients [4–6]. It is common for end of life patients to explore his or her spirituality [7]. However, the lack of agreement on the conceptualisation of spirituality in both research and clinical practice often results in a non-systematic and intuitive approach to patients’ spiritual needs [7–10].

Spirituality can be envisioned as existential and/or religious [8, 11]. Essentially, spirituality is defined as a ‘way individuals seek and express meaning and purpose and the way they experience their connectedness to the moment, to [the] self, to others, to nature and to the significant or sacred’ [12]. Religion can be thought of as an organised system of beliefs, practices and ways of worship [13]. Although religion may be a way to express spirituality, some individuals focus less on the spiritual aspects of religion and more on the traditions, social interactions and rituals [14]. In this way, spirituality and religion are multidimensional concepts that can co-exist in the same framework, but are also considered distinctive phenomena [15–17].

Health beliefs are related to a person’s cultural background and spiritual or religious affiliations [18]. Patients’ perceptions and experiences of illness, care and death are influenced by their culture, values, beliefs, life experiences and perceived meaning in life [15]. In palliative care contexts, illness-related concerns, social or externally mediated factors and psychological and spiritual considerations impact the preservation of one’s sense of dignity for which a sense of autonomy and self-perception emerge as mediators [19, 20].

Autonomy can be related to the capacity for decision-making as well as functional capacity [21]. Furthermore, palliative care patients’ perception of autonomy may be described as a sense of freedom along with having the right to make his or her own decisions [22].

End of life choices may reflect the meaning of life, unfinished business and planning for one’s final days. Palliative care patients’ decision-making may be influenced by religious, spiritual and existential beliefs, expectations, values and experiences, as well as a sense of personal meaning and satisfaction in life. Cultural factors are also inherent because cultural expectations, traditions and personal characteristics impact end of life decisions [23]. Furthermore, as patients near the end of life, the nature and impact of these personal values may change as well as the decisions he or she needs to make [24–27].

The advantages of involving patients in their own care includes improved patient communication, satisfaction and enhanced dignity [28]. However, failure to discuss and understand patient preferences in end of life care may lead to unnecessary pain, suffering and an excessive use of resources [25].

In palliative care, patients face multiple and complex decisions that, together with the physical, psychosocial and spiritual distress that often accompany the life-threatening illness process, can lead to decisional conflict [29, 30] – a state of uncertainty about a specific course of action, feeling uninformed and unsupported, lack of clarity of personal values and beliefs and dissatisfaction with choice [31, 32] – and thus, influence patients’ end of life experience and wellbeing [29]. Therefore, this study aims to explore the influence of spirituality on healthcare decision-making in palliative care outpatients, particularly by analysing patients’ personal perceptions of decisional conflict and the relationship to spiritual wellbeing and, by exploring patients’ perceptions of spirituality and autonomy in healthcare decision-making.

## Methods

A cross-sectional study, quantitative methodology and interpretative description was developed. For the computation of the minimum sample size, the primary outcome considered was the assessment of the correlation between the two scales measuring decisional conflict and spiritual wellbeing administered to the sample. For a medium effect size (d = 0.3), a power of 0.8 and a significance level of 0.05, a minimum of 84 participants is required [33].

A battery of tests was administered to outpatients of two palliative care services from two reference oncology institutes from March 2018 to May 2019, consecutively.

Scoring was performed face-to-face by the same interviewer, in real time, using the assessments, and all questions were read aloud due to educational or health conditions that made it difficult for subjects to fill out paper forms. Patients could choose to be alone or accompanied during the administration. The records did not contain information that could identify the participants.

Participant selection criteria included outpatients from the palliative care programmes, the ability to speak the native language and sufficient cognitive abilities to understand the questions. Subjects in a vulnerable state, or who lacked the ability to understand the administered questionnaires and/or who could not provide informed consent, were excluded.

This study complied with all ethical guidelines for human experimentation stated in the Helsinki Declaration.

For statistical data analysis, the Statistical Package for the Social Sciences (SPSS®) version 25 was used. Descriptive statistics using the median and interquartile range, median [IQR], frequencies and percentages, n (%), were used for analysis of quantitative and categorical variables, respectively. Comparison of quantitative variables between groups was done using non-parametric Mann-Whitney rank sum test. Pearson correlation was computed for normally distributed variables and Spearman correlation otherwise. A significance level of 0.05 was pre-determined. This study is exploratory in nature, hence no adjustment for multiple testing was performed [34].Therefore, some findings could be due to chance.

The analysis of the spirituality themes was influenced by the interpretative phenomenological approach. Median and interquartile range, median [IQR], and percentages, n (%), were used to analyse the semi-structured interview results and compare with the scores of the DCS total score and FACIT-Sp total score and SPS subscale.

### Instruments

#### Informed consent and information document to the participant

All documents made available for the participants complied with the policy of the Ethics Committee of each institution.

#### Sociodemographic questionnaire (SQ)

This survey was designed to collect sociodemographic characteristics - such as gender, age, marital status, education, professional status, household and religion - and clinical information – such as diagnosis, the anatomical location of the disease, time since diagnosis, treatments performed and first palliative care consultation.

#### Semi-structured interview (SSI)

It aimed to collect qualitative and quantitative information about how patients defined their own spirituality, the importance of spirituality during illness, spiritual care, the influence of illness in the sense/meaning of life and the ability to make decisions related to health. This semi-structured interview included closed-ended questions: sense of meaning in life, is spirituality important during illness, did sense/meaning of life change with diagnosis, previously received spiritual support, perceived capacity to make health decisions and early decision-making and if decision-making in health would be facilitated through specialised care that considered one’s values and beliefs. Four short-answer questions were also included: description of one’s spirituality, how is spirituality important during illness, how did sense of meaning in life change with illness and most suitable person to give spiritual support. This interview was performed due to the scarcity of data on the personal experience/meaning and opinion of patients regarding his or her own spirituality and decision-making in this context. It was developed based on a culturally validated end of life questionnaire directed to palliative care patients spirituality [35]. The English translated version is presented in the Supplementary material.

#### Decisional conflict scale (DCS)

This scale was composed of 16 self-completed items and five subscales, scored from 0 (strongly agree) to 4 (strongly disagree). It measures personal perceptions of decisional conflicts of patients in healthcare, namely: a) uncertainty in choosing options; b) factors contributing to uncertainty, such as feeling informed, understanding the benefits and risks and having support in decision-making; c) effective decision-making, such as the perception that the choice was informed and values-based and feeling satisfaction with choice [31, 32]. Total scores range from 0 to 100 and, in both the total score and subscales, a higher score indicates greater decisional conflict. A cut-off point may be used such that scores lower than 25 are associated with implementing decisions, and scores exceeding 37.5 are associated with decision delay or feeling unsure about implementation [36]. A translated and cultural-adapted version already validated was used in this study [31].

Internal consistency was examined in our sample by calculating the Cronbach’s alpha coefficient for the total scale (α = 0.847) and for subscales, ranging from 0.415 to 0.933 (Table 1).

**Table 1:** Internal consistency and descriptive statistics for the DCS (*N = 95*)

	Cronbach’s alpha	Minimum	Maximum	Median	IQR	Range
Uncertainty	0.508	25.00	75.00	41.67	[25.00, 50.00]	0–100
Informed	0.831	0.00	75.00	50.00	[33.33, 75.00]	0–100
Values Clarity	0.933	8.33	75.00	33.33	[25.00, 50.00]	0–100
Support	0.415	16.67	66.67	25.00	[25.00, 41.67]	0–100
Effective Decision	0.470	12.50	68.75	31.25	[25.00, 37.50]	0–100
DCS total score	0.847	16.00	41.00	24.00	[19.00, 30.00]	0–100

*DCS* Decisional Conflict Scale

#### Functional assessment of chronic illness therapy – spiritual well-being (FACIT – Sp)

This scale, which is widely used to measure health-related quality of life, is composed of 39 items. Each item was rated from 0 (not at all) to 4 (very much) in five domains: Physical Well-being (PWB), including reports of physical symptoms; Functional Well-being (FWB), including an analysis of the degree of participation in and experience of daily activities; Social/Familial Well-being (SWB), including an assessment of social support and communication; Emotional Well-being (EWB), including measurement of mood and emotional experience of illness; and Spiritual Well-being (SPS), which comprises Meaning/Peace and Faith subscales [37]. FACIT-Sp was developed to address spiritual wellbeing in a broader sense by focusing on existential aspects and faith. This scale is not limited to religious or spiritual traditions [16, 38]. A translated and cultural-adapted version already validated for Portuguese patients was used in this study [38]. The four domains (PWB, FWB, SWB, EWB) constitute the FACT-G subscale, which represents general quality of life. And, a high score on the FACIT-Sp (PWB, FWB, SWB, EWB and SPS) is considered to correlate with improved wellbeing [39]. In this study, an internal consistency of 0.873 was observed for the total scale, for each subscale it ranged from 0.613 to 0.835 (Table 2).

**Table 2:** Internal consistency and descriptive statistics for the – FACIT-Sp scale (*N = 95*)

	Cronbach’s alpha	Minimum	Maximum	Median	IQR	Range
PWB	0.615	7.00	27.00	18.00	[15.00, 22.00]	0–28
SWB	0.613	8.00	28.00	18.00	[15.17, 20.00]	0–28
EWB	0.762	4.00	23.00	16.00	[12.00, 18.00]	0–24
FWB	0.720	2.00	23.00	13.00	[9.00, 16.00]	0–28
FACT-G	0.813	37.67	92.50	64.17	[56.00, 71.00]	0–108
Meaning/Peace	0.767	8.00	26.00	21.00	[18.00, 24.00]	0–32
Faith	0.725	.00	14.00	9.00	[7.00, 11.00]	0–16
SPS	0.835	8.00	40.00	31.00	[25.00, 34.00]	0–48
TOTAL	0.873	49.00	132.50	93.00	[83.00,104.00]	0–156

*FACIT* – Sp Functional Assessment of Chronic Illness Therapy – Spiritual Well-Being

## Results

The sample was composed of 95 palliative care outpatients. Participants were recruited from palliative outpatient consultations of two Portuguese Institutes of Oncology, namely from the Northern Region (NOI) (*n = 53*) and Central Region (COI) (*n = 42*) (Table 3). Participants selection was made previously, together with the liaison element of the respective institution, which had prior knowledge of each case, in order to select patients who met the desired criteria. All patients approached agreed to participate in the study, but two patients interrupted the administration of the questionnaires due to unexpected pain/symptoms.

**Table 3:** Sociodemographic characteristics

		Oncology Institution		Total *N* = 95
		Northern (NOI) *n* = 53	Central (COI) *n* = 42	
		Median [IQR]	Median [IQR]	Median [IQR]
Age (years)		72 [60,79]	74 [59, 80]	74 [60, 80]
N° family members for support		3 [2, 4]	3 [2, 3]	3 [2, 4]
		n (%)	n (%)	n (%)
Gender	Female	32 (60.4)	16 (38.1)	48 (50.5)
	Male	21 (39.6)	26 (61.9)	47 (49.5)
Marital status	Single	5 (9.4)	5 (11.9)	10 (10.5)
	Married	35 (66)	26 (61.9)	61 (64.2)
	Divorced	3 (5.7)	2 (4.8)	5 (5.3)
	Widow	10 (18.9)	8 (19)	18 (18.9)
	Cohabitation	0	1 (2.4)	1 (1.1)
Location	North region	48 (94.1)	0	48 (51.6)
	Central region	3 (5.9)	40 (95.2)	43 (46.2)
	South region	0	2 (4.8)	2 (2.2)
Education	No studies	11 (20.8)	12 (28.6)	23 (24.2)
	< high school	34 (64.2)	26 (61.9)	60 (63.2)
	High school	6 (11.3)	2 (4.8)	8 (8.4)
	Graduate	2 (3.8)	2 (4.8)	4 (4.2)
Professional status	Retired	39 (73.6)	27 (64.3)	66 (69.5)
	Homemaker	2 (3.8)	3 (7.1)	5 (5.3)
	Employed	1 (1.9)	2 (4.8)	3 (3.2)
	Unemployed	0	2 (4.8)	2 (2.1)
	Disability status	11 (20.8)	8 (19)	19 (20)
Religious	Yes	52 (98.1)	38 (90.5)	90 (94.7)
	No	1 (1.9)	4 (9.5)	5 (5.3)
		*n* = 52	*n* = 38	*n* = 90
Religious practice	Yes	34 (65.4)	15 (39.5)	49 (54.4)
	No	18 (34.6)	23 (69.5)	41 (45.6)
Religion type	Catholic	48 (92.3)	37 (97.4)	85 (94.4)
	Christian	1 (1.9)	0	1 (1.1)
	Jehovah’s Witness	1 (1.9)	1 (2.6)	2 (2.2)
	Spirituality	1 (1.9)	0	1 (1.1)
	UCKG	1 (1.9)	0	1 (1.1)

Forty-eight were female (50.5%), and the median age was 74 years old with a minimum age of 35 and a maximum age of 93. All patients except one reported having family members present for support with a median number of three members (Table 3).

Most participants were married (64.2%), had elementary education (63.2%) and were retired (69.5%). The northern region was represented by 51.6% of the subjects, the central region by 46.2% and the south region by 2.2%. The majority claimed to be religious (94.7%), and 54.4% actively practiced their respective religion. Most patients (94.4%) in this study were catholic (Table 3).

All subjects had received a cancer diagnosis, and the most common type of cancer was gastrointestinal (35.8%). Most participants were submitted to treatments or invasive procedures (84.2%), and the most common treatment was chemotherapy (54.7%). The median follow-up time from diagnosis to the time of admission to the palliative care programme was 21 months (Table 4).

**Table 4:** Clinical Characteristics

		Oncology Institution		Total *N* = 95
		Northern (NOI) *n* = 53	Central (COI) *n* = 42	
		Median [IQR]	Median [IQR]	Median [IQR]
Time since diagnosis until admission in palliative care (months)		21 [14,48]	17.5 [7, 42]	21 [9, 48]
		*n* (%)	*n* (%)	*n* (%)
Diagnosis	Cancer	53 (100)	42 (100)	95 (100)
Cancer Diagnosis	One site	53 (100)	42 (100)	95 (100)
	Two or more sites	3 (5.7)	3 (7.1)	6 (6.3)
Cancer Site	Gastrointestinal	23 (43.4)	11 (26.2)	34 (35.8)
	Head and neck	3 (5.7)	16 (38.1)	19 (20)
	Breast	6 (11.3)	5 (11.9)	11 (11.6)
	Lungs	4 (7.5)	4 (9.5)	8 (8.4)
	Gynaecological	7 (13.2)	1 (2.4)	8 (8.4)
	Prostate	2 (3.8)	3 (7.1)	5 (5.3)
	Colorectal	3 (5.7)	1 (2.4)	4 (4.2)
	Skin	2 (3.8)	1 (2.4)	3 (3.2)
	Bones	2 (3.8)	0	2 (2.1)
	Urinary tract	1 (1.9)	1 (2.4)	2 (2.1)
	Thyroid	1 (1.9)	0	1 (1.1)
	Others	2 (3.8)	2 (4.8)	4 (4.2)
Treatments/Procedures	Yes	47 (88.7)	33 (78.6)	80 (84.2)
	No	6 (11.3)	9 (21.4)	15 (15.8)
Treatment/Procedure Type	Chemotherapy	33 (62.3)	19 (45.2)	52 (54.7)
	Radiotherapy	18 (34)	22 (52.4)	40 (42.1)
	Hormonotherapy	1 (1.9)	1 (2.4)	2 (2.1)
	Immunotherapy	0	3 (7.1)	3 (3.2)
	Surgery	27 (50.9)	13 (31.0)	40 (42.1)
	Tracheostomy	1 (1.9)	4 (9.5)	5 (5.3)
	Others	1 (1.9)	3 (7.1)	4 (4.2)

### Decisional conflict scale

Using a cut-off point of < 25 in the total score for decision implementation, 56 participants (58.95%) were able to implement their decision. Using the total score cut-off of ≥37.5, three subjects (3.16%) were uncertain about their decision.

Positive significant correlations were found among all subscales with the exception of the Support subscale with the Informed and Values Clarity subscales. In other words, in this sample, there was no significant relationship between feeling supported in decision-making and feeling informed and clear about personal values for the benefits and risks/side effects. A weaker but significant relationship between support and effective decision-making was found (Table 5).

**Table 5:** Spearman’s correlations between DCS subscales (*N = 95*)

	Uncertainty	Informed	Values Clarity	Support	Effective Decision
Uncertainty		0.303**	0.382**	0.366**	0.374**
Informed			0.661**	0.077	0.554**
Values Clarity				0.156	0.577**
Support					0.241*

*p < 0.05; **p < 0.01

### FACIT-Sp

Regarding participants’ spiritual wellbeing, the score for the Spiritual Wellbeing (SPS) and Meaning/Peace subscales displayed a significant positive correlation with all FACIT-Sp subscales with the exception of the social wellbeing (SWB) subscale. The Faith subscale showed a significant and positive correlation for the EWB, FWB, FACT-G and Meaning/Peace subscales (Table 6).

**Table 6:** Spearman’s correlations between FACIT-Sp subscales (*N = 95*)

	PWB	SWB	EWB	FWB	Meaning/Peace	Faith	SPS
PWB		0.148	0.455**	0.508**	0.371**	0.113	0.281**
SWB			0.042	0.252*	0.049	0.044	0.040
EWB				0.492**	0.635**	0.382**	0.597**
FWB					0.462**	0.298**	0.446**
FACT-G					0.554**	0.296**	0.494**
Meaning/Peace						0.508**	

*p < 0.05; **p < 0.01

### DCS and FACIT-Sp

Patients who were able to implement their decision (*n* = 56, DCS total score < 25), compared with the remaining 39 participants who scored ≥25, showed significantly higher scores on the EWB (U = 699; *p* = 0.001), FACT-G (U = 792.5; *p* = 0.012), Meaning/Peace (U = 681; *p* = 0.001), Faith (U = 813; *p* = 0.017) and SPS (U = 692.5; *p* = 0.001) subscales, as well as on the FACIT-Sp total scale (U = 755.5; *p* = 0.005). Only 3 patients were unable to implement their decision or were uncertain about their decision (DCS total score ≥ 37.5).

Significant negative correlations were found among several subscales of the DCS and subscales and SPS FACIT-Sp subscale. Higher spiritual wellbeing was found significantly associated with lower levels of uncertainty, feeling informed and supported, higher satisfaction with the choice made and lower decisional conflict (Table 7). Strong significant correlations were also found for higher emotional wellbeing and quality of life and lower levels of uncertainty and decisional conflict (Table 7).

**Table 7:** Spearman’s correlations between FACIT-Sp and DCS scales and subscales

FACIT-Sp	DCS					
	Uncertainty	Informed	Values Clarity	Support	Effective Decision	Total Score
PWB	−0.221*	− 0.10	− 0.081	−0.096	− 0.104	− 0.117
SWB	− 0.044	− 0.058	− 0.066	− 0.038	− 0.096	− 0.078
EWB	− 0.313**	−0.199	− 0.245*	− 0.201	− 0.207*	− 0.307**
FWB	− 0.214*	−0.194	− 0.199	− 0.014	− 0.155	− 0.230*
FACT-G	− 0.265**	− 0.170	− 0.200	− 0.133	− 0.184	− 0.250*
Meaning/Peace	− 0.277**	− 0.250*	− 0.158	− 0.273**	− 0.211*	− 0.287**
Faith	−0.210	− 0.176	− 0.150	−0.191	− 0.263*	−0.275**
SPS	−0.267**	−0.237*	− 0.177	−0.269**	− 0.262*	−0.326**
FACIT-Sp Total	−0.286**	−0.186	− 0.201	−0.190	− 0.224*	−0.284**

*p < 0.05; ** *p* < 0.01

### Semi-structured interview

Data collected during the interviews enabled the analysis of 95 significant statements concerning the description of patients’ spirituality. The analysis was influenced by the interpretative phenomenological approach, in which the researchers made an interpretation of the meaning of the lived experiences of the patients [40]. The authors independently found expressions, which were then compared. This allowed to make theoretical connections, still reflecting the particularity of each experience [40]. This process enabled the authors to reach a total of 16 consensual themes. The most frequent spirituality topic mentioned was ‘Family’, followed by ‘God/Religion’, by 51.6 and 30.5% of the participants, respectively (Table 8).

**Table 8:** Descriptions of patients’ spirituality

Topics	*N* = 95 (%)	Example
Family	49 (51.6)	‘My family’ ‘Family that I love and support’
God/Religion	29 (30.5)	‘God gives me courage’ ‘Jesus Christ is our salvation’
To live	16 (16.9)	‘Will to live’ ‘I think about living’
Faith	11 (11.6)	‘Faith’
Inner strength	11 (11.6)	‘Courage inside me’ ‘To face good and evil’
World	9 (9.5)	‘To see the world’‘society, travel …’
Friends	7 (7.4)	‘To hang out with friends …’ ‘Neighbours …’
Basic needs	7 (7.4)	‘Be able to eat …’ ‘Walk again …’
Work	7 (7.4)	‘My profession, I feel useful’ ‘A working man’
Autonomy	6 (6.3)	‘Have strength to do what I did before’ ‘Have a normal life again …’
Hope	6 (6.3)	‘Have hope’
Healing/Health	5 (5.3)	‘I want to heal myself’‘It’s health!’
Nature	4 (4.2)	‘The mountain air …’ ‘Gardening and taking care of my chickens’
Believe	4 (4.2)	‘To believe’
Suffering	4 (4.2)	‘It’s a sacrifice. I don’t have the will to live. The faster this is settled, the better’ ‘I would like to join my husband, he passed away less than a year ago …’
Superior forces	3 (3.2)	‘Some superior force …’ ‘Destiny, I have this, and I have to face it’

This research aimed to study the relationship between spirituality and patient decision-making. Thus, the median scores of the SPS FACIT-Sp subscale, DCS and FACIT-Sp from the groups that answered ‘yes’ or ‘no’ in the semi-structured closed-ended questions were analysed. The ‘don’t know’ group wasn’t comprised given the small number of answers.

Participants who asserted the importance of spirituality during their illness process (*n* = 85, 89.5%), mainly due to strength (*n* = 22, 25.9%) and support (*n* = 20, 23.5%), indicated higher scores of spiritual wellbeing (median [IQR] = 32 [26, 34] vs 23 [15, 29]) and quality of life (97 [85, 106] vs 80.4 [57.8, 98]), and lower decisional conflict (24 [19, 30] vs 31 [19, 37]) (Table 9).

**Table 9:** Median scores of the DSC, FACIT-Sp and SPS subscale according to participants’ answers on the close-ended questions on the semi-structured interview

Semi-structured interview		DCS total scoreMedian [IQR]	SPS FACIT-SpMedian [IQR]	FACIT-Sp total scoreMedian [IQR]
1. Importance of spirituality during illness	yes (*n* = 85)	24 [19, 30]	32 [26, 34]	97 [85, 106]
	no (*n* = 6)	31 [19, 37]	23 [15, 29]	80.4 [57.8, 98]
2. Sense of meaning in life	yes (*n* = 64)	23.5 [19, 29]	32 [28.5, 35]	100 [90, 108]
	no (*n* = 22)	28 [18, 32]	20 [18, 30]	74.2 [64.7, 85]
3. Sense of meaning in life changed with illness	yes (*n* = 70)	24 [19, 31]	30 [25, 34]	90.3 [80.7, 4101.7]
	no (*n* = 25)	23 [20, 27]	32 [30, 35]	101 [93.8, 111]
4. Spiritual support	yes (*n* = 23)	20 [17, 28]	34 [30, 36]	99 [90.7, 106]
	no (*n* = 72)	24 [20, 31]	30 [23, 33.5]	92.5 [80.5, 102.5]
5. Capacity of decision-making	Own decisions (*n* = 64)	23.5 [19, 29.5]	32 [26.5, 34]	97.5 [86.5, 105.5]
	Dependent on others (*n* = 31)	24 [19, 32]	30 [22, 34]	90.7 [81, 101.7]
6. Capable of early decision-making	yes (*n* = 62)	22 [19, 30]	32 [26, 35]	97 [85, 105]
	no (*n* = 31)	25 [19, 32]	30 [22, 32]	91 [81, 101.7]
7. Important to have spiritual support	yes (*n* = 58)	22.5 [19, 30]	31 [26, 34]	93.4 [86, 106]
	no (*n* = 22)	23.5 [19, 32]	32.5 [26, 35]	98.5 [88, 105]
8. Facilitation of decision-making in health through a specialised care	yes (*n* = 64)	24 [19, 30]	32 [26, 34]	97.7 [88.5, 106.2]
	no (*n* = 21)	24 [19, 31]	30 [25, 35]	95.5 [77, 104]

‘Sense of meaning in life’ was noted by most subjects (*n* = 64, 67.4%); however, 22 participants (23.2%) stated that they did not have a sense of meaning in life, and nine participants (9.5%) did not know if their lives still had any meaning. Considerably higher scores of spiritual wellbeing (32 [28.5, 35] vs 20 [18, 30]) and quality of life (100 [90, 108] vs 74.2 [64.7, 85]) were found for participants who had a sense of meaning in life, as well as lower levels of decisional conflict (23.5 [19, 29] vs 28 [18, 32]) (Table 9).

Most participants (*n* = 70, 73.7%) stated that their sense/meaning of life changed with illness, mainly because of physical limitations (*n* = 34, 48.6%) and the loss of autonomy (*n* = 33, 47.1%). Although the differences in score were not very prominent, patients whose sense of meaning in life didn’t change with the illness presented higher quality of life scores (101 [93.8, 111] vs 90.3 [80.7, 101.7]) (Table 9).

The majority of participants stated not receiving any spiritual care or support during their illness (*n* = 72, 75.8%). Moreover, patients indicated that the most suitable people to provide this kind of support were family members (*n* = 29, 40.3%) particularly, a spouse (*n* = 11, 37.9%); healthcare professionals (*n =* 11, 15.3%), such as psychologists (*n* = 4, 36.4%) and medical doctors (*n* = 3, 27.3%); Gods/saints (*n* = 10, 13.9%); a spiritual/religious community (*n* = 5, 6.9%); informal caregivers/friends (*n =* 2, 2.8%) and oriental therapies e.g. reiki (*n =* 1, 1.4%). Three participants (4.2%) responded that no one could give them support, and eight participants (11.1%) did not know what/who could be the most suitable support system.

On the other hand, 23 subjects (24.2%) had received spiritual support primarily from their spiritual/religious community (*n* = 17, 73.9%), family members (*n* = 4, 17.4%), specifically a spouse (*n =* 1, 25%), and nurses (*n =* 1, 4.3%). These subjects presented higher scores on spiritual wellbeing and quality of life for the SPS (34 [30, 36] vs 30 [23, 33.5]) and FACIT-Sp (99 [90.7, 106] vs 92.5 [80.5, 102.5]), as well as lower decisional conflict score in the DCS (20 [17, 28] vs 24 [20, 31]). Moreover, 58 participants (61.1%) asserted the importance of receiving spiritual care, 22 participants (23.2%) answered that they did not need this kind of care, and 15 (15.8%) indicated ‘didn’t know’. Nevertheless, there were no relevant differences found in the DCS total score, FACIT-Sp total score and SPS subscale for the ‘yes’ and ‘no’ groups (Table 9).

With regard to the perceived capacity to make health decisions, 64 participants (67.4%) claimed to be able to decide on their own, and 31 (32.6%) indicated feeling dependent on others to make health decisions. Concerning early decision-making about their future, 62 participants (65.3%) felt capable, 31 (32.6%) did not feel able and two (2.1%) did not know whether they were fit to engage in early decision-making. Participants who felt capable of making their own decisions and of early decision-making presented slightly higher scores of spiritual wellbeing and quality of life and lower scores on decisional conflict (Table 9).

Most participants (*n* = 64, 67.4%) indicated that decision-making in health would be facilitated through specialised care that considered one’s values and beliefs. On the other hand, 21 participants (22.1%) did not consider it to be relevant, and 10 (10.5%) did not know if it would be pertinent for decision-making. Subjects who found decision-making enabled through specialised care, scored slightly higher on the FACIT-Sp total score (97.7 [88.5, 106.2] vs 95.5 [77, 104]) (Table 9).

## Discussion

This study aimed to explore the relationship between spirituality and decision-making in palliative care outpatients at two oncology institutes (*n* = 95). Participants’ perceptions of decision-making, in specific decisional conflict, and the connection to spirituality, particularly spiritual wellbeing, were analysed.

The DCS showed acceptable reliability and validity when used to assess decision-making related to end of life care [41]. In this study, good internal consistency was observed for both the total scale and subscales; with the exception of subscales Uncertainty, Support and Effective Decision, which displayed low internal consistency. During decision-making, uncertainty can be resolved through information-gathering; however, as a consequence, patients can perceive the decision as effective despite experiencing uncertainty [42]. The FACIT-Sp, which is one of the most common instruments used in a palliative care setting to measure quality of life in general and spiritual wellbeing in particular [6], displayed sufficient to very good internal consistency.

A homogeneous sample of men and women was obtained, with a median age of 74 years old, a minimum age of 35 and a maximum age of 93. Studies have shown that increasing age was significantly associated with better physical, psychological and existential scores [43, 44] as well as better end of life preparation [45]. On the other hand, spiritual and quality of life outcomes are usually worse for younger patients due to the possibility of a shortened lifespan, functional deterioration and unfulfilled aspirations, which can be sources of great spiritual and psychosocial distress [46].

The majority of patients claimed to be religious, mainly catholic, in which more than half practiced their respective religion. Faith, rituals and associated prayers are also shown to influence many areas of patients’ lives [47–49], such as positive emotions, quality of life and social interactions [47, 48, 50] as well as death, disability, financial problems and one’s future in as part of family [48, 50–53]. Positive religious coping is correlated with faith, which promotes health and psychological adaptation to serious illness and provides a positive spiritual support system [54–56]. The social support that results from religious engagement is a key factor, since this support system is found within faith communities and also in local neighbourhoods and service communities [57, 58].

However, when patients rely solely on a higher power for mental and physical health without any problem-solving mechanisms, it may result in poorer long-term health outcomes [53, 59]. A negative religious coping mechanism (e.g., perceiving illness as a punishment or abandonment by God) is associated with higher levels of distress, confusion and depression, in addition to a poorer sense of physical and emotional wellbeing and quality of life [60, 61]. Furthermore, the need to conform to a particular belief system for health decision-making and a family’s concerns related to religious beliefs or the importance of religious faith may result in anxiety and negative effects on health. These factors may also exacerbate the effect of family stressors on depression [62–64].

The impact of spirituality on the overall wellbeing of patients was observed in this study. Patients’ who found spirituality important during illness presented higher scores of spiritual wellbeing, quality of life, and lower levels of decisional conflict. In addition, having a sense of meaning in life was associated with higher levels of quality of life and spiritual wellbeing, and lower levels of decisional conflict. Thus, considering spirituality during care is beneficial for terminally ill patients because it allows the evaluation of patients’ spiritual wellbeing or to what extent patients’ spirituality can help them make sense of their lives in order to feel whole, hopeful and peaceful [16, 65, 66].

The main effect of illness on a patient’s sense of meaning in life was mainly related to physical limitations and loss of autonomy. Patients whose sense of meaning in life did not change with illness displayed a better score of quality of life. Palliative care patients valued being able to perform daily tasks and feeling useful when appropriate [21]. Patients who feel independent may present higher levels of optimism, self-esteem, social support and spiritual wellbeing, in addition to fewer symptoms of depression [16, 67]. Late referrals to palliative care are an aspect that may complicate patient involvement in care and the promotion of autonomy. In late engagement, besides the reduced ability of the patient to participate in decision-making due to advanced illness, the decisions that remained mainly concern terminal symptom management [68].

Furthermore, significant associations among greater spiritual wellbeing and better physical, emotional and functional wellbeing, higher levels of meaning/peace and faith and a better quality of life were found. The influence of spiritual wellbeing on psychological and physical dimensions has been enhanced in most studies [51, 69–71]. These results highlighted the importance of spirituality as a coping mechanism [59]. The social dimension did not show an association with spiritual wellbeing as observed in other studies [16, 72].

Patients’ views on spirituality were found to be connected with family as well as related to their religious beliefs. These connections provided participants various benefits, such as meaning and purpose in life, compassion toward others and a positive spiritual influence [73–75]. Of the topics that assessed spirituality descriptions, ‘Family’ and ‘God/religion’ were the most commonly mentioned topic. The major role of one’s spirituality in illness as mentioned by patients was strength and support. Furthermore, family members were described by patients as being the best-suited to provide spiritual support, followed by healthcare professionals (namely, psychologists and medical doctors). The multidimensionality of spirituality was identified as a unique and individual dimension that may be related to the self, others and the world. It can be moved by the search for meaning in life [12]. The relationship between religion and spirituality should be noted, but the roles they play may be distinct [15–17].

A relationship between spiritual wellbeing and decisional conflict was observed in this study. Higher levels of spiritual wellbeing were significantly associated with less uncertainty, feeling more informed and supported, greater satisfaction with decisions and less decisional conflict. Furthermore, strong significant associations were found between higher emotional wellbeing and quality of life and lower levels of uncertainty and decisional conflict. According to O’Connor [32], decisional conflict along with cognitive, emotional and social factors can increase a person’s perception of uncertainty, which difficult decision-making. Decisional conflict may be expressed by the verbalisation of uncertainty, indecision between choices, delayed decision-making and questioning personal values and beliefs while attempting to make a decision [42]. Besides, patients in this context may feel fearful that their decisions may bring them closer to death [76].

Studies point to the impact of patients’ spirituality on end of life decision-making and show that high levels of spiritual wellbeing associate with improved quality of life, disease coping, adjustment to diagnosis, the ability to cope with symptoms and protect against depression, hopelessness and the desire for a hastened death [11, 47]. This way, spirituality may help both clinicians and researchers with clinical conceptualisation and subsequent treatment planning [16, 65, 66] given that participants who received spiritual care presented lower scores of decisional conflict and higher levels of spiritual wellbeing and quality of life. Patients who were able to implement their decisions displayed higher levels of emotional and spiritual wellbeing, greater levels of meaning/peace, more faith and a better quality of life. Patients found the decision-making process satisfactory if the process was supportive and informed such that their decision-making was consistent with their values and beliefs [41]. In this sense, spiritual wellbeing was helpful to patients coping with end of life decisions because it likely facilitated decision-making through positive coping mechanisms [77], promoting a peaceful death experience and addressing the needs of dying patients [16, 78].

The perception of making a good or bad decision may be dependent on a person’s perception of illness and their global beliefs, such as identity, health and overall goals, level of distress related to his or her illness and spirituality [79]. This variance may impact the decision-making balance associated with benefits and risks, which may lead to doubt [76]. Healthcare professionals need to determine how the patient prefers to be included in decision-making process given that these issues are often culturally based with spiritual or religious implications [18]. Discussion of a patient’s care plans (such as advanced care planning or end of life care) and the acknowledgement of the patient’s specific demographic and clinical characteristics may help predict patients’ spiritual beliefs and attitudes. These actions may provide an opportunity for patients to explore decisions with their families, enable clearer lines of communication with healthcare professionals and develop patient-centred goals [18, 27, 43].

Most participants considered it important to have spiritual care, however the majority didn’t receive spiritual care. The lack of preparation and education on spirituality at the end of life may result in discomfort and, therefore, avoidance of many healthcare professionals to broach these issues [18]. It is important to promote knowledge of the importance of spirituality and how this type of support/intervention may enhance spirituality as a means of comfort and peace during life-threatening illnesses [16, 78].

There is a need to be familiar and respectful of traditions and beliefs and, if possible, integrate them into the care plan. A specialised care that takes into consideration one’s values and beliefs was found to enable health decision-making. Thus, the need for healthcare professionals to impartially assess the needs of patients as a whole by identifying a person’s values and belief system, spiritual history, distress and needs is essential for promoting dignity, autonomy and the right for the patient’s self-determination [80]. As well as taking into account the specific cultural and ethical background of patients, advocacy will provide individualised and appropriate care for palliative care patients [81].

## Conclusions

According to the principles of palliative care, which aim to improve patients’ and their families’ quality of life through the prevention and relief of pain and physical, psychosocial and spiritual suffering [5], patient dignity and autonomy are central points for improving care [22]. Autonomy is defined as the ‘right of a capable person to decide his/her own course of action' [27], and has been identified as one of the four major ethical principles [82].

Most participants in this study felt capable of decision-making in healthcare situations as well as of early decision-making. An autonomous decision presupposes the capacity to decide, and the capacity to value one’s own existence is essential for assigning personhood and moral agency [83]. The benefits of autonomy in health are reflected in the form of physical, psychological, social and spiritual wellbeing [21].

Spirituality is considered to be a unique part of everyday interactions and life [18] and it was mostly described by participants through family or God/religion. This way, it is denoted the multidimensional characteristics of spirituality. Family plays an important role in end of life search for meaning [84]. It is seen as the unit of care since it finds its own potential and enables achievements at the end of life [84]. In addition, family support that can help advocate for the patient, may decrease stress and provide end of life comfort, even if patients have the capacity to make their own health decisions [85].

Several patients related their spirituality to God/religion. Nevertheless, when beliefs are not used in an adaptive way, decision-making could be affected. Thus, palliative care teams should be alerted to signs of negative religious coping and collaborate with spiritual care workers to address the distress experienced by these patients [60].

Spirituality plays an important role at the end of life. In fact, spiritual wellbeing was associated with higher rates of physical, emotional and functional wellbeing and a better quality of life.

The influence of spiritual wellbeing on decision-making was evident, as participants with higher levels of spiritual wellbeing showed less uncertainty with choice, feelings of being more informed and supported, a higher level of satisfaction with decisions and less decisional conflict. Furthermore, patients who were able to implement their decisions displayed better emotional and spiritual wellbeing and an improved quality of life. Thus, it is important to consider end of life decisions in terms of patients’ values, beliefs and relief of suffering [23].

Given the effects of end of life care and the difficulty and uncertainty associated with patient’s choices, there is a need for methods to measure the quality of those choices [41]. Since palliative patients prefer a person-centred, individualised care approach and responsive healthcare professionals who consider their requests in counselling and treatment [86], specialised care that considers one’s values and beliefs may improve overall health decision-making, as indicated by participants.

An autonomous decision indicates that patients were able to re-evaluate their lives and accept their decreasing health status and imminent death [76]. Therefore, healthcare professionals should consider spirituality as a potentially important component of every patient’s physical wellbeing and mental health [87]. Specifically, they should become aware of how patients use their spirituality and/or faith to cope with their illness, which can help identify whether specialised mental health and/or religious practices would be beneficial for pain management [59, 88] while promoting patients’ self-determination and empowerment [86]. This would enable to restore a sense of dignity, autonomy and self-worth in patients [89].

This exploratory study raises several pertinent hypotheses which should be further validated in new studies based on independent datasets [34].

To analyse the qualitative data, the interpretative phenomenological analysis process should be conducted, to allow a more in-depth, comprehensive and narrative analysis of the results [40]. Furthermore, the semi-structured interview should be validated for the Portuguese palliative population.

In this study, the clinical data collected was based on patients’ perception of their diagnosis, treatments and health status. Nevertheless, future studies should comprise the medical record of the patient, such as diagnosis and stage of the illness, criteria for palliative care transition, Palliative Performance Scale (PPS) and treatment plan. Furthermore, patient selection was made previously by a liaison element, according to the selection criteria and patients’ availability, e.g. ambulance transportation schedules. Future studies should comprise all patients and record the information of the patients approached, so comparisons can be made on the differences between the patients who participate and those who do not, either due to refusal or due to the illness condition.

Moreover, future studies should explore the optimal way to address patients’ spirituality and needs with respect to their clinical history [60]. Future studies should also explore the effects of promoting patients decision-making based on each person’s unique perspective, taking into account that this process is influenced by their psychophysiological state, personal and/or professional values and beliefs, spiritual and sociocultural beliefs, needs, education and knowledge [86]. This way, the need to involve an advocate or other health professional with special expertise in cross-cultural issues and spirituality is essencial [18]. This approach will help address patients as a whole by respecting their autonomy, dignity and end of life process.

## Supplementary information

**Additional file 1.** Semi-structured interview

## Data Availability

The datasets used and/or analysed during the current study are available from the corresponding author on reasonable request.

## Abbreviations

COI: Portuguese Institute of Oncology – Central region
DCS: Decisional Conflict Scale
EWB: Emotional Well-being subscale
FACIT-Sp: Functional Assessment of Chronic Illness Therapy – Spiritual Well-Being
FACT-G: General Quality of life subscale
FWB: Functional Well-being subscale
NOI: Portuguese Institute of Oncology – Northern region
PWB: Physical Well-being subscale
SPS: Spiritual Well-being subscale
SQ: Sociodemographic Questionnaire
SSI: Semi-structrured Interview
SWB: Social Well-being subscale
