# Supplementary material for: The influence of spirituality on decision-making in palliative care outpatients: a cross-sectional study
Source: BMC Palliat Care. 2020 Feb 21;19:22. doi: 10.1186/s12904-020-0525-3 (PMC7035674; doi:10.1186/s12904-020-0525-3)
Supplement: Supplementary file 1 — Additional file 1. Semi-structured interview [file 12904_2020_525_MOESM1_ESM.docx]

**Semi-structured interview**

1. How do you describe your own spirituality?

_____________________________________________________________________________

1. Is spirituality important during the illness process?

Yes No Don’t know

If so, how: ____________________________________________________________________

1. Do you feel that you have a sense of meaning in life?

Yes No Don’t know

1. Has your sense of meaning in life changed since the diagnosis? If so, how?

_____________________________________________________________________________

1. Have you had any kind of spiritual support during the illness process?

Yes No Don’t know

If so, what kind of support and provided by whom? _____________________________________________________________________________

If you answered no, or feel it wasn’t enough, who do you believe could provide this kind of support?

_____________________________________________________________________________

1. Do you feel: capable of making your own decisions

dependent on other people to make decisions

1. Do you feel capable of early decision-making (e.g. making a will)?

Yes No Don’t know

1. Considering the definition of spirituality as a personal dimension and a process of self-discovery, expressed by the search for the meaning of life (example: God, family, nature, art ...). Do you believe it would be important for you to have spiritual support?

Yes No Don’t know

1. Do you believe that decision-making in health would be facilitated through a specialised care, which respects the values ​​and beliefs of each patient?

Yes No Don’t know
